# Attachment of Ferredoxin: NADP^+^ Oxidoreductase to Phycobilisomes Is Required for Photoheterotrophic Growth of the Cyanobacterium *Synechococcus* sp. PCC 7002

**DOI:** 10.3390/microorganisms10071313

**Published:** 2022-06-29

**Authors:** Xiying Li, Chenhui Huang, Peijun Wei, Kun Zhang, Chunxia Dong, Qing Lan, Zhenggao Zheng, Zhengdong Zhang, Jindong Zhao

**Affiliations:** 1 State Key Laboratory of Protein and Plant Genetic Engineering, School of Life Science, Peking University, Beijing 100871, China; huangchh@shsmu.edu.cn (C.H.); wei_peijun@pku.edu.cn (P.W.); zk1898@pku.edu.cn (K.Z.); dongcx@pku.edu.cn (C.D.); a7lan9001@163.com (Q.L.); zhengzhenggao@pku.edu.cn (Z.Z.); zhangzhd2017@pku.edu.cn (Z.Z.); 2 School of Medicine, Shanghai Jiao Tong University, Shanghai 200011, China

**Keywords:** cyanobacteria, FNR, NDH, photoheterotrophic growth, phycobilisome

## Abstract

Two types of cyanobacterial phycobilisomes (PBS) are present: the hemidiscoidal PBS (CpcG-PBS) and the membrane-bound PBS (CpcL-PBS). Both types of PBS have ferredoxin:NADP^+^ oxidoreductase (FNR) attached to the termini of their rods through a CpcD domain. To date, the physiological significance of the attachment remains unknown. We constructed a mutant (dF338) which contains an FNR lacking the N-terminal CpcD domain in *Synechococcus* sp. PCC 7002. Isolated CpcG-PBS from dF338 did not contain FNR and the cell extracts of the mutant had a 35 kDa protein cross-reacting to anti-FNR antibodies. dF338 grows normally under photoautotrophic conditions, but little growth was observed under photoheterotrophic conditions. A *cpcL* (*cpcG2*) mutant grows extremely slowly under photoheterotrophic conditions while a *cpcG* (*cpcG1*) mutant, in which PBS rods could not attach to the cores of the CpcG-PBS, can grow photoheterotrophically, strongly suggesting that the attachment of FNR to CpcL-PBS is critical to photoheterotrophic growth. We show that electron transfer to the plastoquinone pool in dF338 and the *cpcL* mutant was impaired. We also provide evidence that trimeric photosystem I (PSI) and intact CpcL-PBS with a full-length FNR is critical to plastoquinone reduction. The presence of a NADPH-dehydrogenase (NDH)-CpcL-PBS-PSI trimer supercomplex and its roles are discussed.

## 1. Introduction

Cyanobacteria are a large group of prokaryotes that perform oxygenic photosynthesis, and they play important roles in global carbon and nitrogen cycles. Phycobilisomes (PBS) are supramolecular pigment–protein complexes composed of phycobiliproteins and linker proteins and they are the major antenna complexes for light energy harvesting in cyanobacteria [1,2,3,4,5]. There are two types of PBS in most cyanobacteria: hemidiscoidal PBS and CpcL-PBS. The structures of several hemidiscoidal PBS have been determined recently [5] and they all consist of peripheral rods and a central core. A peripheral rod usually has several hexamers joined together through the linker protein CpcC, which contains a Pfam00427 domain and a Pfam01383 domain [5,6,7]. The attachment of a peripheral rod to the core is accomplished by the linker protein CpcG1 [8]. The CpcG1-containing PBS is herein called CpcG-PBS. CpcG1 has a Pfam00427 domain at its N-terminus and an extension with α-helices at its C-terminus [7,9]. The Pfam00427 domain of CpcG1 is located in the cavity of the proximal hexamer of the rods and the C-terminal helices interact with the core. At the distal end of the peripheral rod is another linker protein, CpcD, which contains a Pfam01383 domain and is responsible for distal termination of PBS rod extension [10]. The CpcL-PBS was characterized more recently [11,12] and it consists of only rod that is attached to the thylakoid membrane by the linker protein CpcL. The N-terminal portion of CpcL is homologous to CpcG1 and the genes encoding CpcL were earlier annotated as *cpcG2* (in *Synechococcus* sp. PCC 7002 and *Synechocystis* sp. PCC 6803) or *cpcG3* (in *Anabaena* sp. PCC 7120) [13]. The C-terminal portion of CpcL contains a hydrophobic tail that anchors the CpcL-PBS to the thylakoid membranes [11,12]. The CpcL-PBS is preferentially associated with photosystem I (PSI) and the light energy absorbed by CpcL-PBS is transferred mainly to PSI [11,12,14,15,16]. Although CpcG-PBS is more closely associated with photosystem II (PSII) [9,17,18], the light energy absorbed by CpcG-PBS can be transferred to either PSII or PSI and distribution of the absorbed light energy between the two photosystems are regulated by a process called state transitions [19,20,21].

In linear photosynthetic electron transfer, water is oxidized by PSII and the electrons are transferred through electron transfer chain to the final electron acceptor NADP^+^ and the last reaction is carried out by ferredoxin:NADP^+^ oxidoreductase (FNR) [22]. FNR in plant chloroplasts has a two-domain structure: a FAD-binding domain and a NAD-binding domain, and these two domains are responsible for electron transfer from ferredoxin to NADP^+^ [23,24]. The FNR from cyanobacteria also contains the two domains of plant’s FNR and carries out the same biochemical reactions in photosynthetic electron transfer. However, it additionally contains a CpcD domain [25], which belongs to Pfam01383 protein family [26]. Due to the presence of the CpcD domain at its N-terminus, some cyanobacterial FNR is attached to rods of CpcL-PBS as well as of CpcG-PBS [27,28,29]. However, the physiological roles of the attachment of FNR to PBS remain unknown.

Even though there is only one gene, the *petH* gene, encoding FNR in cyanobacteria, two isoforms of FNR are observed: the full-length FNR (FNR_L_) that has three domains and a short version of FNR (FNR_S_) that lacks the CpcD domain [25]. Analysis of *petH* translation reveals that different translational start points are responsible for the generation of the two isoforms in cyanobacteria [30,31]. Based on enzymatic activity and mutant phenotypes, it was postulated that in cyanobacteria, the FNR_L_ mainly has a role in reduction of NADP^+^, whereas the FNR_S_ is mainly involved in cyclic electron transfer (CET) [30,31,32]. Although the electron transfer route in CET in both cyanobacteria and plant chloroplasts is not well understood at the moment [33], it has been firmly established that NADPH dehydrogenase complex (NDH) is critical to CET in both cyanobacteria and higher plants [34,35,36,37] and the NDH-PSI supercomplex formation is key to its functions [38,39]. The formation of NDH-PSI supercomplex in cyanobacteria has also been demonstrated and it requires participation of the CpcL-PBS [40]. The presence of a cytochrome *b*_6_*f* complex (cyt *b_6_f*)-bound FNR in chloroplasts suggests a possible route of a direct electron transfer from FNR to cyt *b_6_f* complex [41] although a cyt *b_6_f* complex with a bound FNR has not been observed in cyanobacteria.

Here we report that a truncated FNR without the CpcD domain was unable to attach to PBS and the strain with the mutant gene was unable to grow photoheterotrophically. We provide evidence that the CET in the cyanobacteria is impaired when FNR is not associated with PBS. We provide evidence that the CET in the cyanobacteria is impaired when FNR is not associated with PBS and demonstrate that FNR_L_ plays an important role in CET in *Synechococcus* 7002.

## 2. Materials and Methods

### 2.1. Culture Conditions and Mutant Construction

The wild-type and mutant strains of *Synechococcus* sp. PCC 7002 were grown in A^+^ medium [42] at 37 °C with illumination of white fluorescent light at a light intensity of approximately 200 μmol photons m^−2^ s^−1^. Green light condition was provided with LED light with a wavelength at 520 nm at an intensity of 50 μmol photons m^−2^ s^−1^. The cultures were bubbled with air plus 1% CO_2_. When needed, kanamycin was added to the media at a concentration of 50 μg mL^−1^, and erythromycin of 25 μg mL^−1^. Glycerol (10 mM) and DCMU (10 μM) were used when cells were grown photoheterotrophically. Iron deficiency cultures were grown according to Alcántara-Sánchez et al. [43]. Cell density was determined by measuring optical density at 730 nm.

The deletion mutant of *cpcG2* (ΔcpcG2) was constructed as described [14]. The strain ΔcpcC (deletion of the *cpcC* gene) was constructed according to Zheng et al. [5]. The strain ΔpsaL was constructed as described [44]. The strain ΔcpcG1 (deletion of the *cpcG1* gene) was constructed as described [45]. The strain ΔcpcBA, which lacks the *cpcBA* genes, was constructed according to Huang et al. [46]. The strain dF338, in which the *petH* gene was replaced by *petH2D* encoding FNR_S_, was constructed as follows. A 1000 bp fragment from 643 bp upstream to 357 bp downstream of the first ATG codon of the *petH* gene was amplified by PCR with primer pairs of P1/P2. DNA fragments encoding kanamycin resistance cassette (Kan^r^), the *cpcBA* promoter and *petH2D* gene (and truncated *petH* gene for SLF8) were amplified by PCR with primer pairs of P3/P4, P5/P6 and P7/P8. With two-way fusion PCR methods [47], the resultant PCR products were transformed to wild-type strain and the transformants were selected on 50 μg mL^−1^ kanamycin. The transformants were screened by PCR with primer pairs of P9/P10 for complete replacement of the *petH* gene by the *petH2D* gene. The same strategy was used for construction of strain SLF8, in which a mutant *petH* encodes a mutant FNR with a deletion from Glu75 to Pro95 in its amino acid sequence. The primers used in the construction of the mutant *petH* for SLF8 were P11/P12 and P8/P13. To generate pku-F2d-oe strain, DNA fragments encoding the *cpcBA* promoter, a mutant *petH* encoding FNRs and kanamycin resistance cassette (Kan^r^) were amplified by PCR with primer pairs of P6/P14, P7/P15, and P16/P17. The PCR fragments were fused by two-way fusion PCR followed by insertion into a chromosome docking site between 2588743–2588744. Fragments upstream and downstream of this site were amplified with the primer pairs of P18/P19 and P20/P21 for homologous recombination. The primers used in this study are listed in Supplementary Table S1.

### 2.2. Protein Extraction and Immunoblotting

Thylakoid membranes were isolated as previously described [48]. After removal of unbroken cells, the supernatant containing thylakoid membranes and soluble proteins were separated after centrifuged at 20,000× *g* for 30 min at 4 °C. Soluble proteins were precipitated for sample preparation. PBS were prepared according to previous work [5,9]. PBS from sucrose density gradient centrifugation were desalted with ultrafiltration using Milipore Amicon Ultra centrifugal filters. PSI was isolated according to previous work [15,49] for EM analysis. Proteins were separated with 15% sodium dodecyl sulfate-polyacrylamide gel electrophoresis (SDS-PAGE). Proteins in the gel were transferred to a nitrocellulose membrane for immunoblotting analysis and the antibodies against FNR from cyanobacteria were used as primary antibodies, which were detected by the secondary antibodies from Promega (Beijing, China).

N-terminal sequences of proteins were determined by sequencing with a protein sequencer ABI491.

### 2.3. Fluorescence Measurements

Fluorescence emission spectra at 77 K and room temperature fluorescence were obtained following the methods in the previous work [14,50]. Post-illumination rise of Chl fluorescence was measured as described previously using Dual-PAM-100 instrument [48].

### 2.4. P700 Measurement

P700 of PSI was measured using a Dual-PAM-100 instrument (Heinz Walz, Effeltrich, Germany) as described [21]. Cells were harvested and adjusted to OD_730nm_ of 3.0 and were dark-adapted for 5 min before turning on the actinic light. The wavelengths of the actinic lights at 635 nm and 440 nm were used. DCMU at a concentration of 10 μM was added to culture when needed.

## 3. Results

### 3.1. The CpcD Domain Is Responsible for Association of FNR with PBS

**Figure 1:**
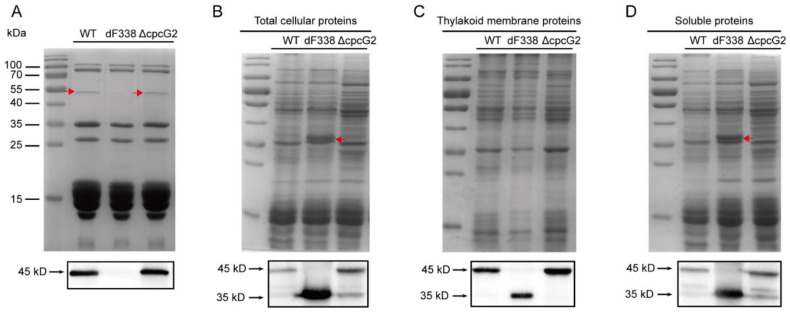
SDS-PAGE and immunoblotting analysis of ferredoxin:NADP^+^ oxidoreductase (FNR) from different strains. (**A**) SDS-PAGE separation of phycobilisome (PBS) proteins from the wild-type (WT), dF338 and ΔCpcG2. Red arrows indicate FNR protein, which is missing in the dF338 mutant. The molecular mass markers are shown as bars on the left side. Proteins separated by SDS-PAGE were transferred from gel to nitrocellulose membrane for immunoblotting and a portion of the blot is shown in the box below the gel image. (**B**–**D**), total cellular proteins, thylakoid membrane proteins and soluble proteins were extracted and separated by SDS-PAGE respectively, from the strains WT, dF338 and ΔCpcG2. The red arrows in panel (**B**,**D**) indicate an increased intensity of the protein bands at the position of approximately 35 kDa. Immunoblotting analysis of FNR proteins are shown in the boxes below each gel. The molecular masses of the immunoblotting bands are shown on the left of the boxes as indicated by black arrows.

To study roles of FNR attachment to PBS, we constructed a mutant strain of *Synechococcus* 7002 which produces only FNR_S_ (strain dF338). Because deletion of *petH* is lethal in cyanobacteria, the *petH* gene of *Synechococcus* 7002 was replaced by a modified version of *petH*, named *petH2D*, that had a deletion from residue 1 to residue 95. The correct mutation was confirmed (Figure S1). PBS isolated from the wild-type strain (WT), dF338 and ΔCpcG2, which is a mutant lacking *cpcG2* and its CpcL-PBS is missing while its CpcG-PBS remain unchanged [14], were analyzed with SDS-PAGE. Protein bands with a molecular mass of 45 kDa could clearly be visualized by Commassie brilliant blue staining in the lanes with the isolated CpcG-PBS from WT and ΔCpcG2 while it was not present in CpcG-PBS from dF338 strain. The bands from WT and ΔCpcG2 were cross-reacted with the anti-FNR antibodies in immunoblotting (Figure 1A). The 45 kDa bands were further analyzed by N-terminal sequencing and they had a sequence of MYGITSTANSTGNQSYAN, confirming that they were FNR. In total cellular extracts, FNR_L_, the 45 kDa bands were found to cross-react with the anti-FNR antibodies in WT and ΔCpcG2. In dF338, only a 35 kDa protein was detected by the immunoblotting (Figure 1B), confirming that the CpcD domain of FNR was deleted in the mutant. The proteins from the cell extracts were separated into membrane and soluble fractions and they were again probed with immunoblotting after SDS-PAGE. As shown in Figure 1C,D, FNR_L_ is present in the both membrane and soluble fractions from the WT and ΔCpcG2 but not in the dF338’s fractions. It also shows that FNR_S_ is present in WT, dF338 and ΔCpcG2. In the soluble fractions, there were more FNR_S_ than the membrane fractions in WT and ΔCpcG2 and there were two bands detected by immunoblotting, indicating that two isoforms of FNR_S_ could be present in soluble fractions in WT and ΔCpcG2. It should be noted that we used a *cpcBA* promoter for the expression of the *petH2D* gene in dF338 and the amount of FNR in dF338 is higher than that in the WT. We have not been able to generate a mutant strain that contains a truncated form of *petH* with its own promoter.

### 3.2. The Strain dF338 Is Able to Perform State Transitions

**Figure 2:**
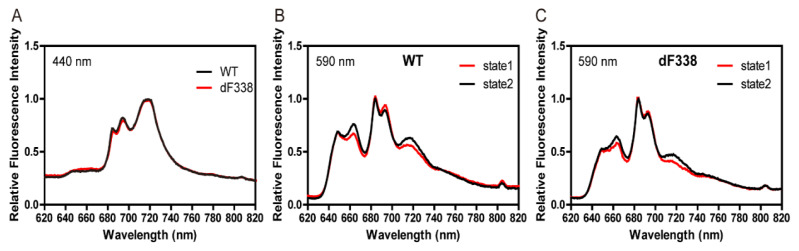
77 K fluorescence spectra of the wild-type (WT) and dF338. (**A**) Emission spectra of WT (black line) and dF338 (red line) in state 2 conditions were collected with an excitation light at 440 nm. (**B**,**C**) 77 K fluorescence emission spectra of WT and dF338, respectively, were obtained with an excitation light at 590 nm. Cells were brought to state 1 by illuminating with blue light with DCMU (red line) and to state 2 with incubation in the dark (black line) before frozen in liquid nitrogen.

Since CpcG-PBS are mostly associated with PSII [17] while FNR plays a major role in electron transfer of PSI, it was first predicted that the attachment of FNR to PBS may have a role in state transitions. We first measured 77 K fluorescence emission spectra of WT and dF338 with a chlorophyll-absorbing (440 nm) excitation light (Figure 2A) and found that they were nearly identical, indicating the that PSII to PSI stoichiometry of dF338 was the same as that of WT. The 77 K fluorescence emission spectra were then measured with PBS-absorbing light under state I or state II conditions and the results suggested that the state transitions in dF338 strain were not impaired (Figure 2B,C). Room temperature fluorescence inductions in the presence of DCMU also showed that the state transitions of dF338 were not impaired (Figure S2). It has been shown that impairing state transitions leads to slower growth under green light that is preferentially absorbed by PBS [21]. We therefore measured the growth rates of dF338 under a green light and found that they were almost identical to that of the WT (Figure S3), suggesting that the major role of the attachment of FNR to PBS is unlikely related to state transitions. Room temperature fluorescence inductions and growth curve under green light conditions were also measured for ΔcpcG2 (Figures S2C and S3). The results for ΔcpcG2 were almost identical as that of wild-type, demonstrating that CpcL-PBS are not involved in state transition.

### 3.3. The Strain dF338 Is Impaired of Photoheterotrophic Growth

**Figure 3:**
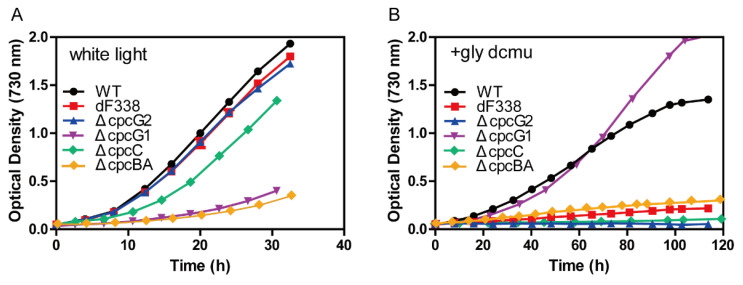
Growth curves of the wild-type (WT) and the mutant strains. (**A**) Photoautotrophic growth curves of WT and the mutant strains were obtained under white light condition provided with cool white fluorescent light at 200 μmol photons m^−2^ s^−2^. (**B**) Photoheterotrophic growth curves of WT and the mutant strains in the presence of glycerol (10 mM) and DCMU (10 μM) under a light intensity of 50 μmol photons m^−2^ s^−2^. Colors of the curves are as follows: black for WT; red for dF338; blue for ΔcpcG2; purple for ΔcpcG1; green for ΔcpcC; and yellow for ΔcpcBA.

We measured growth of dF338 and several other strains under photoautotrophic and photoheterotrophic conditions. While the growth of the strains dF338 and ΔcpcG2 under photoautotrophic conditions with white light was very similar to that of WT (Figure 3A), their growth under photoheterotrophic conditions (10 mM glycerol and 10 μM DCMU) was much slower than that of WT (Figure 3B). The two other strains, ΔcpcBA and ΔcpcG1, were also measured for their growth. ΔcpcBA lacks the genes encoding the α- and β-subunits of phycocyanin and no PBS rods can be synthesized. ΔcpcG1 lacks CpcG1 and the peripheral rods, which can be synthesized, could but cannot be attached to the cores (Figure S4A). Therefore, the mutated CpcG-PBS in ΔcpcBA and ΔcpcG1 were the similar in that they have no rods attached to the cores and therefore there would be no FNR attached to their CpcG-PBS. Both ΔcpcBA and ΔcpcG1 grew much slower than WT under photoautotrophic conditions (Figure 3A) because of insufficient light absorption due to a reduced light-absorbing cross-section sizes. However, their growth under photoheterotrophic conditions was different: the growth rates of ΔcpcG1 was slightly faster than that of WT while ΔcpcBA could hardly grow (Figure 3B). Since the CpcL-PBS in ΔcpcBA could not be synthesized and it is not affected by the deletion of *cpcG1* in ΔcpcG1, these results were suggestive that CpcL-PBS is more critical to the normal growth under photoheterotrophic conditions. We used another mutant ΔcpcC to further test this possibility. The strain ΔcpcC lacks the gene *cpcC* encoding the rod linker and the rods of both CpcG-PBS and CpcL-PBS contain only one hexamer [5] (Figure S4A). As shown in Figure 3A, photoautotrophic growth of ΔcpcC was slower than that of WT but significantly faster than that ΔcpcG1 and ΔcpcBA. Under photoheterotrophic conditions, little growth of ΔcpcC was observed (Figure 3B). Importantly, the attachment of FNR to the CpcC-less CpcG-PBS was confirmed by SDS-PAGE analysis (Figure S4B).

### 3.4. Photosynthetic Electron Transfers

**Figure 4:**
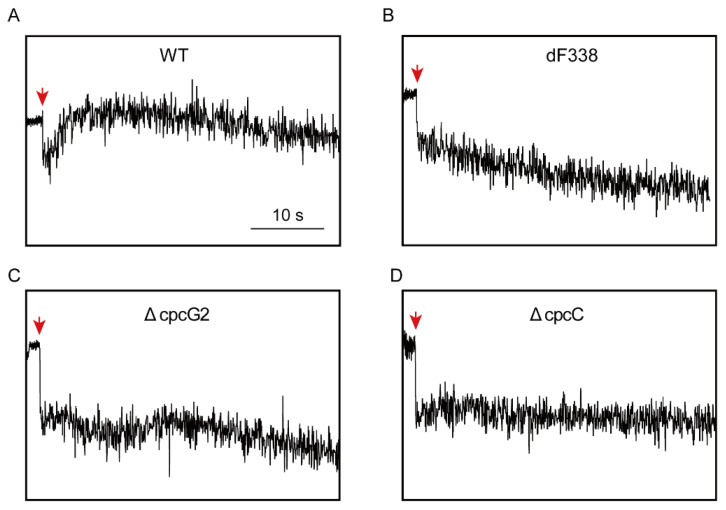
Post-illumination rise of Chl fluorescence (PIRF) analysis. PIRF analysis of the wild-type (WT) (**A**), dF338 (**B**), ΔcpcG2 (**C**) and ΔcpcC (**D**). Cells were illuminated with an actinic light for 30 s before the actinic light was turned off (downward arrows) and the transient increase of Chl fluorescence at the wavelength of 685 nm was monitored. Bar indicates 10 s.

To understand why attachment of FNR_L_ to CpcL-PBS is required for growth under photoheterotrophic conditions, post-illumination rise of Chl fluorescence (PIRF), which is attributable to the reduction of plastoquinone (PQ) [38,40,41,51], was measured. WT exhibited significant increases of PIRF when the actinic light was turned off (Figure 4A). However, PIRF was not observed in dF338, ΔcpcG2 and ΔcpcC (Figure 4B–D), suggesting that these mutants were impaired of electron donation to PQ pool. When WT and dF338 cultures were exposed to a strong light (1300 μmol m^−2^ s^−1^), dF338 showed a faster decrease of oxygen evolution rate than WT while its recovery from photoinhibition was slower than that of WT (Figure S5).

**Figure 5:**
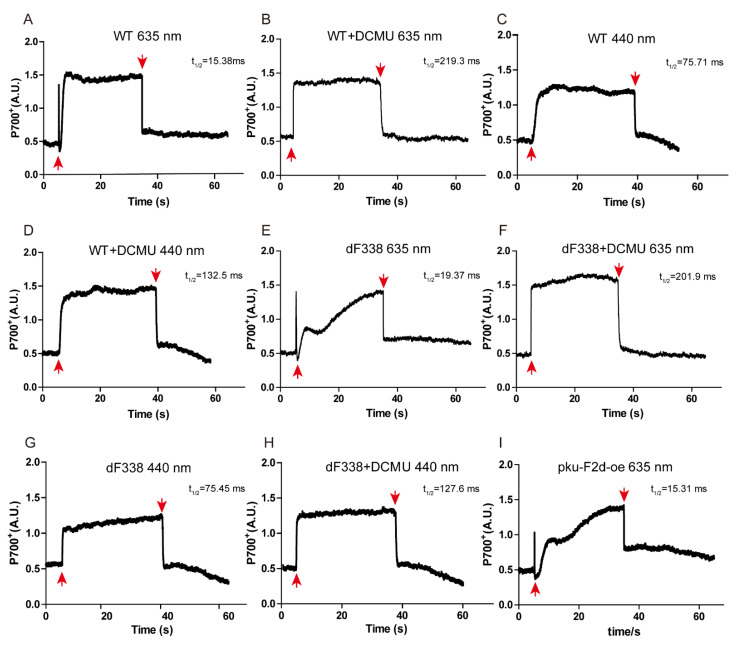
Kinetic analyses of P700 photooxidation and reduction of P700^+^. Strains of WT (panels **A**–**D**), dF338 (panels **E**–**H**) and pku-F2d-oe (the strain that overexpresses the *petH2D* gene, panel **I**) were incubated in the dark for 5 min before the actinic lights at 635 nm (panels **A**,**B**,**E**,**F**,**I**) or at 440 nm (panels **C**,**D**,**G**,**H**) were switched on. When needed (panels **B**,**D**,**F**,**H**), DCMU was added at a concentration 10 μM before the dark incubation. The upward and downward red arrows in the panels indicate turning on or turning off of the actinic lights, respectively.

The P700 redox kinetics of WT and dF338 were measured and it was found that the rates of P700^+^ reduction in dF338 were comparable to the rates of P700^+^ reduction in WT under the conditions tested (Figure 5). The P700 redox change induced with a PBS-absorbing (635 nm) light in WT cells are shown in Figure 5A. When the light was turned on, a spike of P700 signal was immediately observed, indicating a fast oxidation and a reduction, followed by a slower oxidation to reach an oxidized steady state. When the light was turned off, the P700^+^ was rapidly reduced with a half time of 10–20 ms. In the presence of DCMU, which blocks electron transfer from PSII to PQ pool, the initial spike of P700 signal disappeared and the reduction of P700^+^ was much slower with a half time of approximately 200 ms when the light was turned off (Figure 5B). The initial spike of the P700 signal observed in Figure 5A was also absent if the actinic light was a Chl-absorbing light at 440 nm (Figure 5C), which is preferentially used by PSI. For the strain dF338, while the initial spike of P700 signal is observed, the following oxidation of P700 was much slower (Figure 5E). The slower oxidation of P700 is abolished when DCMU was added (Figure 5F) or the actinic light was Chl-absorbing light (440 nm) (Figure 5G). The very slow oxidation of P700 observed in Figure 5E was likely a result from overexpression of the *petH2D* gene because overexpression of the *petH2D* gene in a WT background (strain pku-F2d-oe) also led to a similarly slow oxidation of P700 (Figure 5I). It is also possible that the strain with overproduced FNRs could have a faster electron donation to the PQ pool as observed in *Synechocystis* [52,53], leading to a slower oxidation of P700 (Figure 5E).

### 3.5. Association of CpcL-PBS with PSI

**Figure 6:**
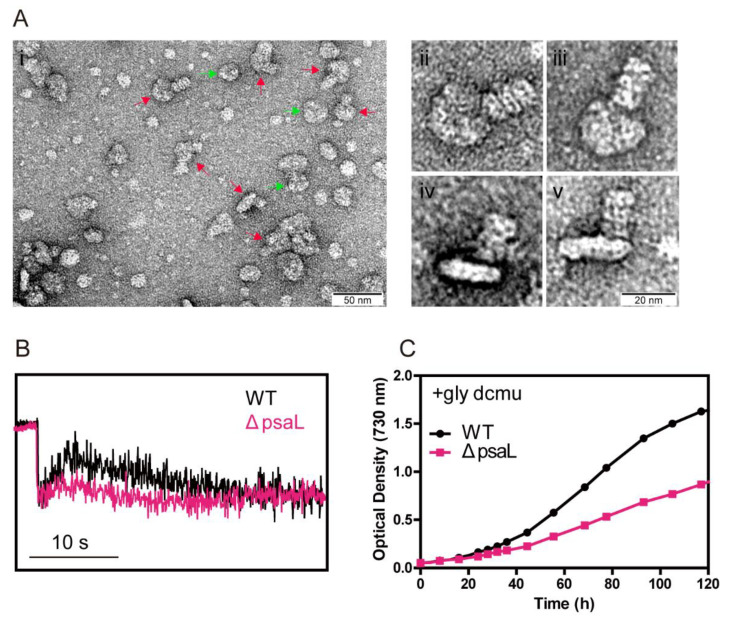
EM analysis of CpcL-PBS-photosystem I (PSI) complexes from *Synechococcus* 7002 and phenotypic characterization of ΔpsaL mutant. (**Ai**) Electron microscopic images of the negatively stained particles of the trimeric PSI (green arrows) and CpcL-PBS-PSI trimer complexes (red arrows). Bar indicates 50 nm. Panels (**Aii**) through (**Av**) show enlarged images of top view (**Aii**,**Aiii**) and side view (**Aiv**,**Av**) of individual complexes of CpcL-PBS-PSI trimer complexes. Bar indicates 20 nm. (**B**) PIRF analysis of the wild-type (WT) (black trace) and ΔpsaL (pink trace). (**C**) Photoheterotrophic growth of WT (black) and ΔpsaL (pink) in the presence of 10 mM glycerol and 10 μM DCMU under light intensity of 50 μmol photons m^−2^ s^−2^.

Based on an earlier study that CpcL-PBS was associated with PSI in the filamentous cyanobacterium *Anabaena* 7120 [15], we searched conditions for isolating CpcL-PBS-PSI complexes in *Synechococcus* 7002. We were unable to obtain CpcL-PBS-PSI complexes from WT and other strains under normal growth conditions. However, under the conditions that increase CET, such as low iron in growth media [43], we were able to isolate CpcL-PBS-PSI complexes with ultracentrifugation in high phosphate-sucrose buffer. As shown in Figure 6A(i), the isolated particles of PSI trimer and CpcL-PBS-PSI trimer complexes can be observed by EM with negative staining. Enlarged images of some particles are shown in Figure 6A(ii–v). The interactive site for CpcL-PBS association with PSI trimer seemed to be the interface between PSI monomers (Figure 6A(ii,iii)). From the side view, the CpcL-PBS rods stood upwards, but the angles between PSI timer and the rods were variable (Figure 6A(iv,v)). We did not observe CpcL-PBS-PSI monomer complexes with negatively stained electron microscopy. Measurement of PIRF revealed that ΔpsaL, a *psaL* mutant that cannot form PSI trimer [44], had a much lower signal in PIRF (Figure 6B), suggestive of an impairment of electron transfer to PQ pool. Under the photoheterotrophic growth conditions, the *psaL* mutant grew more slowly than WT (Figure 6C) but faster than dF338. Under photoautotrophic conditions, the growth rate of the *psaL* mutant was nearly identical to that of WT (Figure S6).

**Figure 7:**
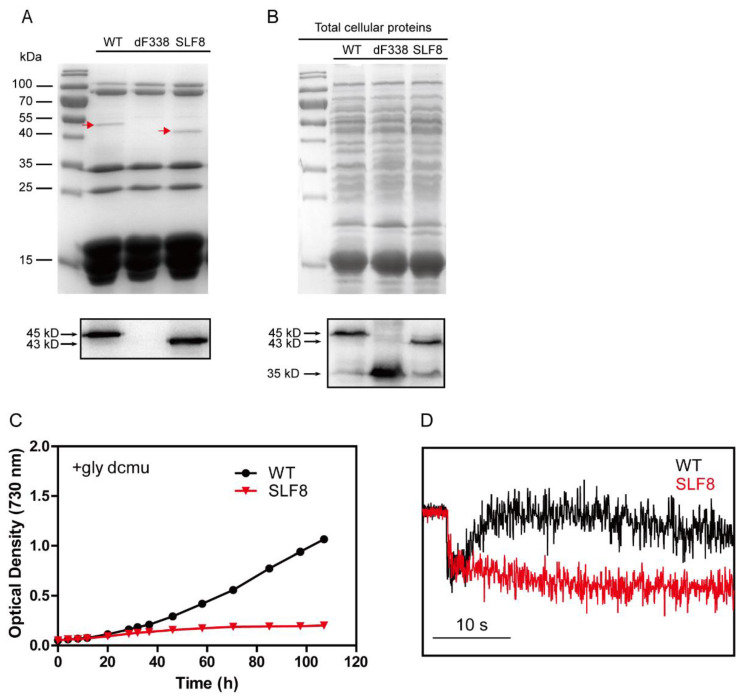
Characterization of the mutant strain SLF8. (**A**) PBS of the wild-type (WT), dF338 and SLF8 strains were isolated and the components were separated with SDS-PAGE. Red arrows indicate FNR protein bands, which was smaller in the lane from SLF8. Immunoblotting with FNR antibodies was performed and result is shown in the lower box with molecular masses on the left side. (**B**) Total cellular proteins from the same strains in panel A were separated by SDS-PAGE and stained with Coomassie blue. Immunoblotting with FNR antibodies was performed and result is shown in the lower box with molecular masses on the left side. In both gels, the left lanes contain molecular mass markers. (**C**) Photoheterotrophic growth of WT (black) and SLF8 (red) in the presence of 10 mM glycerol and 10 μM DCMU. (**D**) Analysis of electron donation to the PQ pool in WT (black) and SLF8 (red) by PIRF. Bar indicates 10 s.

As mentioned earlier, the CpcD domain of FNR_L_ is linked to the other two domains through a flexible linker region. To gain more insight into the mechanism of FNR_L_ in photoheterotrophic growth, we constructed a strain (SLF8) which has a mutant FNR with a shortened linker region. The region from 75 to 95 residues within the flexible region of FNR_L_ was deleted (Figure S7) and this mutant FNR (FNR_Ls_) has a molecular mass of 43 kDa. It should be noted that the flexible region of FNR_L_ is not conserved among the cyanobacterial FNR_L_ [30] and a previous work did not find post-translational modification in residues 75–95 in this region of FNR_L_ in *Synechococcus* 7002 [54]. Analysis of isolated PBS from the strain SLF8 with SDS-PAGE showed that the band at 45 kDa position in WT was missing in the lane of SLF8 and a new band was present at the position of 43 kDa, which cross-reacted with the anti-FNR antibodies (Figure 7A), demonstrating FNR_Ls_ was able to attach to PBS. Immunoblotting of the total cellular extracts (Figure 7B) showed there were two forms of FNR in SLF8, a 43 kDa protein and a 35 kDa protein, suggesting the mutant SLF8 is able to synthesize FNR_S_ as WT does. SLF8 was able to grow normally under photoautotrophic conditions (Figure S6) but could not grow under photoheterotrophic conditions (Figure 7C), and little PIRF was observed (Figure 7D). It is worthwhile to note that even though we used the *cpcBA* promoter for the expression of shortened *petH* gene in SLF8, the mutant FNR_Ls_ was less abundant than in dF338.

## 4. Discussion

The cyanobacterial FNR contains an additional CpcD domain compared to the plant FNR and association of the cyanobacterial FNR_L_ with PBS was discovered three decades ago [25]. The cyanobacterial *petH* gene encoding FNR_L_ is also responsible for the formation of the plant-type FNR, the two domain FNR or FNR_S,_ which is generated by translation from the second translation start position [30,31] and it is located mainly in cytoplasm [43]. There are two types of PBS in most of the cyanobacteria, CpcG-PBS and CpcL-PBS, and they both harbor FNR_L_ [29]. Even though the association of FNR_L_ with PBS is expected to play important physiological roles as the FNR_L_-PBS association is conserved in nearly the entire phylum of cyanobacteria, the functions of the attachment of FNR_L_ to PBS remained mysterious. In this study, we report the construction of the mutant strain dF338, in which the gene *petH* encoding FNR_L_ was replaced by a mutant *petH* gene, the *petH2D*, which encodes FNR_S_. The inability for FNR_S_ to attach to PBS (Figure 1) does not affect dF338’s growth rate under normal growth conditions (Figure 3) and dF338 was able to perform state transitions as compared with WT (Figure 2 and Figure S2). However, it did affect dF338’s photoheterotrophic growth (Figure 3) when PSII is inhibited by the PSII inhibitor DCMU and the electron source is NADPH generated by oxidation of externally added glycerol. Therefore, the growth experiments strongly suggested that electron donation from NADPH to PQ pool is impaired in dF338.

A set of mutant strains that change PBS structures besides dF338 (Figure S4A) provided clues for understanding why attachment of FNR_L_ to PBS is needed for photoheterotrophic growth. Among these mutants, dF338 is the only mutant that has no FNR_L_ and the other mutants affect the attachment of FNR_L_ to either CpcG-PBS or CpcL-PBS in different ways. In ΔcpcG1, no FNR_L_ could be attached to the rod-less CpcG-PBS while its CpcL-PBS is unchanged. Its normal photoheterotrophic growth (Figure 3B) suggests that the attachment of FNR_L_ to CpcG-PBS is not required for PQ pool reduction by NADPH. The evidence provided by the photoheterotrophic growth (Figure 3B) and PIRF (Figure 4), which is widely used in studies of the reduction of PQ pool by NDH [40,55], from the strains ΔcpcG2 (no CpcL-PBS), ΔcpcBA (no rods of any PBS) and dF338 collectively shows that the attachment of FNR_L_ to CpcL-PBS is critical to the electron donation to PQ pool from NADPH and its function could not be replaced by CpcG-PBS.

It has been shown previously that CpcL-PBS is important for energy transfer from PBS to PSI [11,12,14,16] and it forms the CpcL-PBS-PSI complexes in *Anabaena* 7120 [15]. Here, we provide evidence that CpcL-PBS could interact with PSI trimer and forms a CpcL-PBS-PSI complexes in *Synechococcus* 7002 (Figure 6A). In plant chloroplasts, PSI could interact with NDH to form an NDH-PSI supercomplex and a recently determined structure of NDH-PSI supercomplex shows that LCH of PSI is key to the formation of supercomplex [38,39]. Although the NDH-PSI supercomplex has not been isolated from the cyanobacteria, its existence has been demonstrated in *Synechocystis* 6803 and its CpcL-PBS is required for the NDH-CpcL-PBS-PSI supercomplex formation [40]. The results in Figure 6 strongly suggest that PSI oligomer formation could be important to the supercomplex formation in *Synechococcus* 7002 because the photoheterotrophic growth of a PSI monomer mutant is significantly slower than that of WT and its PIRF is much smaller. Similar phenotypes have been observed in the *psaL* mutant of *Anabaena* 7120, which could not form PSI tetramer [48]. The roles of CpcL-PBS in photoheterotrophic growth are further studied with the strain ΔcpcC, in which both CpcG-PBS and CpcL-PBS have only one hexamer layer on their rods. Although no CpcL-PBS has been isolated from this strain, the CpcG-PBS was isolated from ΔcpcC and it has FNR_L_ attached (Figure S4B). It is expected that the only hexamer layer CpcL-PBS from ΔcpcC should also have FNR_L_ attached. Yet, ΔcpcC grows very poorly under photoheterotrophic conditions and has little PIRF (Figure 3B and Figure 4D), strongly suggesting that the full-length CpcL-PBS is needed for proper electron donation to PQ pool from NADPH.

**Figure 8:**
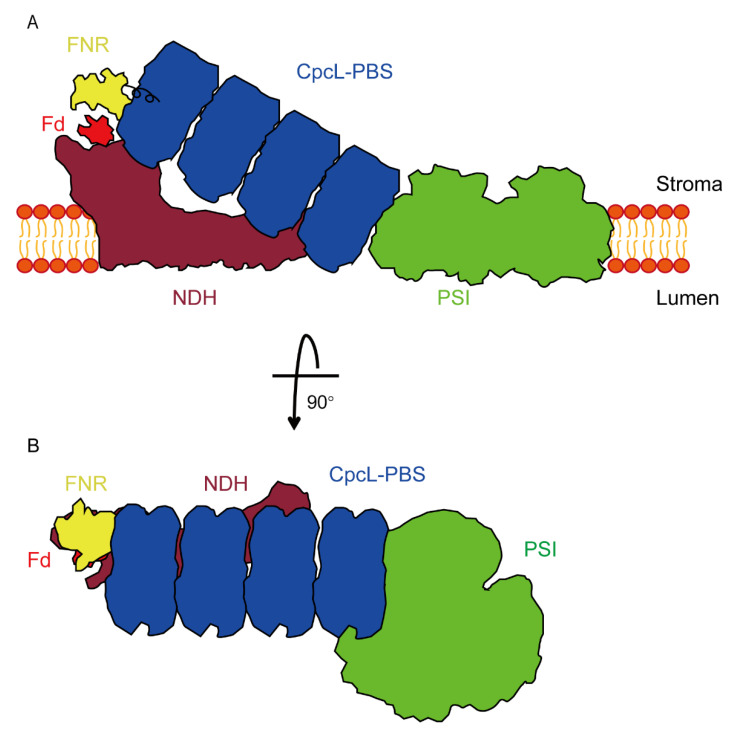
Schematic model of NADPH-dehydrogenase complex (NDH)-CpcL-PBS-PSI trimer supercomplex from sideview (**A**) and top-view (**B**). CpcL-PBS-PSI trimer complex interacts with NDH to form supercomplex. In the supercomplex, the FNR attached to CpcL-PBS could efficiently transfer electrons from NADPH to Fd that is in turn oxidized by NDH located on thylakoid membranes under photoheterotrophic growth conditions. The model is drawn in proportion according to the actual sizes of the supercomplex components.

Based on the previous evidence and our current study, we propose a model for the cyanobacterial supercomplex of NDH-CpcL-PBS-PSI trimer (Figure 8). In this model, CpcL-PBS is important not only in connecting PSI trimer with NDH, but also in delivering the attached FNR_L_ to the proper position of NDH so that more efficient electron transfer could be performed from NADPH to PQ pool. The position of FNR_L_ is critical to the supercomplex’s function because a shortened flexible region between the CpcD domain and the other two domains in FNR_Ls_, which could be attached to PBS, led to poor photoheterotrophic growth and low PIRF (Figure 7).

Several types of NDH exist in the cyanobacteria and they play different roles in various cellular processes [34,35,56]. Although the mechanisms of CET in the cyanobacteria and chloroplasts are not completely understood at the moment [33], accumulated evidence has clearly demonstrated that NDH plays a major role in cyanobacterial CET [35,57,58,59]. Besides NDH, FNR_S_ could also be important to CET around PSI and it has been suggested that interaction of FNR_S_ with cyt *b_6_f* complex [41] could play a critical role in this process. Under photoheterotrophic growth conditions, no electron from PSII could be transferred to the PQ pool and the ultimate reductant source for growth is sugar that generate NADPH for ATP generation through electron transfer chain shared by respiration and photosynthesis. The roles of NDH under these conditions are therefore two folds: (i) it could directly participate CET by receiving electrons from reduced ferredoxins from PSI directly [36,37,60,61] and (ii) it uses reduced ferredoxin generated from NADPH through FNR to reduce PQ pool. It is evident that CET would eventually lose all its electrons without NADPH-generated ferredoxin as electron source under heterotrophic conditions because the electron transfer in CET is not 100% efficient and loss of electron is unavoidable. Our experimental results show that the attachment of FNR_L_ to CpcL-PBS is needed for optimal electron transfer activity from NADPH to PQ pool and this process is likely performed by the NDH-CpcL-PBS-PSI supercomplex. It should be noted that all mutants reported in our study remained alive for more than a week under photoheterotrophic conditions and their rates of P700^+^ reduction were not significantly changed (Figure 5). Therefore, it is possible that FNR-dependent reduction of ferredoxin from NADPH could occur in cyanobacterial cytosol, which in turn reduce PQ pool through NDH and its capacity and efficiency are the key factors for photoheterotrophic growth. For *Synechococcus* 7002, they are not high enough to sustain a normal photoheterotrophic growth. Similarly, a *psaL* mutant of *Anabaena* 7120 showed little PIRF and its N_2_-fixing ability was largely diminished [48]. On the other hand, the inability of FNR to attach to PBS in *Synechocystis* 6803 did not affect its reduction of PQ pool from NADPH significantly [30]. It was also reported that a *cpcG2* mutant lacking CpcL-PBS in *Synechocystis* 6803 could grow photoheterotrophically, but its PIRF was significantly reduced [40].

The roles of the attachment of FNR_L_ to CpcG-PBS remain unclear and more work is needed in the future. It is our speculation that attachment of FNR_L_ to CpcG-PBS might play a role in organization of PSII and PSI on thylakoid membranes. More work is needed to understand how each component of the supercomplex interacts with the others and how the electron transfers in the supercomplex are regulated.

## 5. Conclusions

Besides energy transfer from phycobiliproteins to PSI, CpcL-PBS are important to NDH-dependent electron transfer to the PQ pool in *Synechococcus* 7002 and this function could be carried out by a NDH-CpcL-PBS-PSI trimer supercomplex. The attachment of FNR_L_ with a functional flexible region to an intact CpcL-PBS is required for its optimal activity.

## Data Availability

Data can be found within article and in Supplementary Materials.

## Supplementary Materials

The following supporting information can be downloaded at: https://www.mdpi.com/article/10.3390/microorganisms10071313/s1, Figure S1: PCR confirmation of the construction of the dF338 mutant. Figure S2: Room temperature fluorescence inductions of the wild-type (WT), dF338 and ΔCpcG2. Figure S3: Growth curves of the wild-type (WT) and mutant strains under green light condition. Figure S4: Schematic representations of CpcG-PBS and CpcL-PBS in different strains. Figure S5: Photoinhibition and recovery of wild-type (WT) and dF338. Figure S6: Growth curves of strains under photoautotrophic condition. Figure S7: Molecular characterization of wild-type (WT) and SLF8. Table S1: Sequences of the oligonucleotides primers.
