# Supplementary material for: Attachment of Ferredoxin: NADP+ Oxidoreductase to Phycobilisomes Is Required for Photoheterotrophic Growth of the Cyanobacterium Synechococcus sp. PCC 7002
Source: Microorganisms. 2022 Jun 29;10(7):1313. doi: 10.3390/microorganisms10071313 (PMC9319322; doi:10.3390/microorganisms10071313)
Supplement: Supplementary file 1 [file microorganisms-10-01313-s001.zip › microorganisms-1773657-supplementary.pdf]

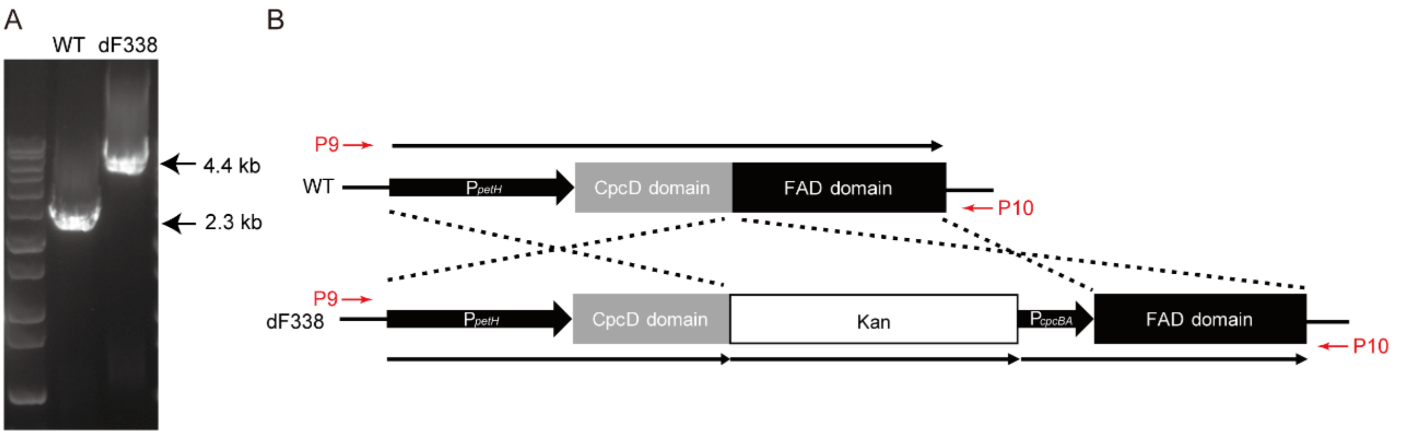

Supplementary Figure S1. PCR confirmation of the construction of the dF338 mutant. (A) PCR confirmation of strain dF338 construction. Chromosomal DNA of the wild-type (WT) and dF338 strains were isolated and used as templates for PCR and the amplified fragments from these strains are indicated by arrows. (B) Schematic drawing of dF338 construction. The mutant was confirmed by PCR with primer pairs of P9/P10.

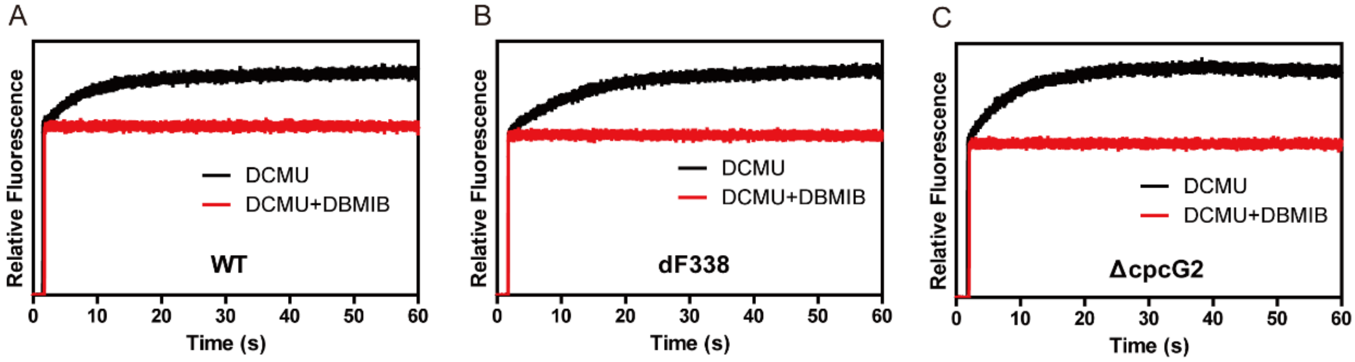

Supplementary Figure S2. Room temperature fluorescence inductions of the wild-type (WT), dF338 and ΔcpcG2. Cells from WT (A), dF338 (B) and ΔcpcG2 (C) were incubated in the dark for 5 min before actinic light was switched on and fluorescence emission at 685 nm was recorded. DCMU (10 μM, black traces) or DCMU/DBMIB (10 μM/10 μM, red traces) were added to cell suspension before dark incubation.

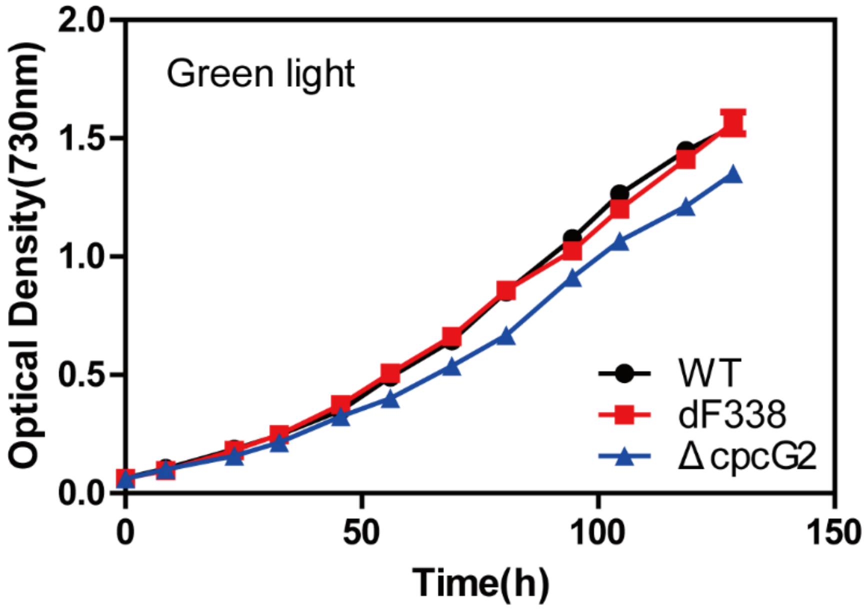

Supplementary Figure S3. Growth curves of the wild-type (WT) and mutant strains under green light condition. WT (black), dF338 (red) and ΔcpcG2 (blue) were grown under green light at 50 μmol photons m^{-2} s^{-2}. Green light condition was provided with LED light with a wavelength at 520 nm.

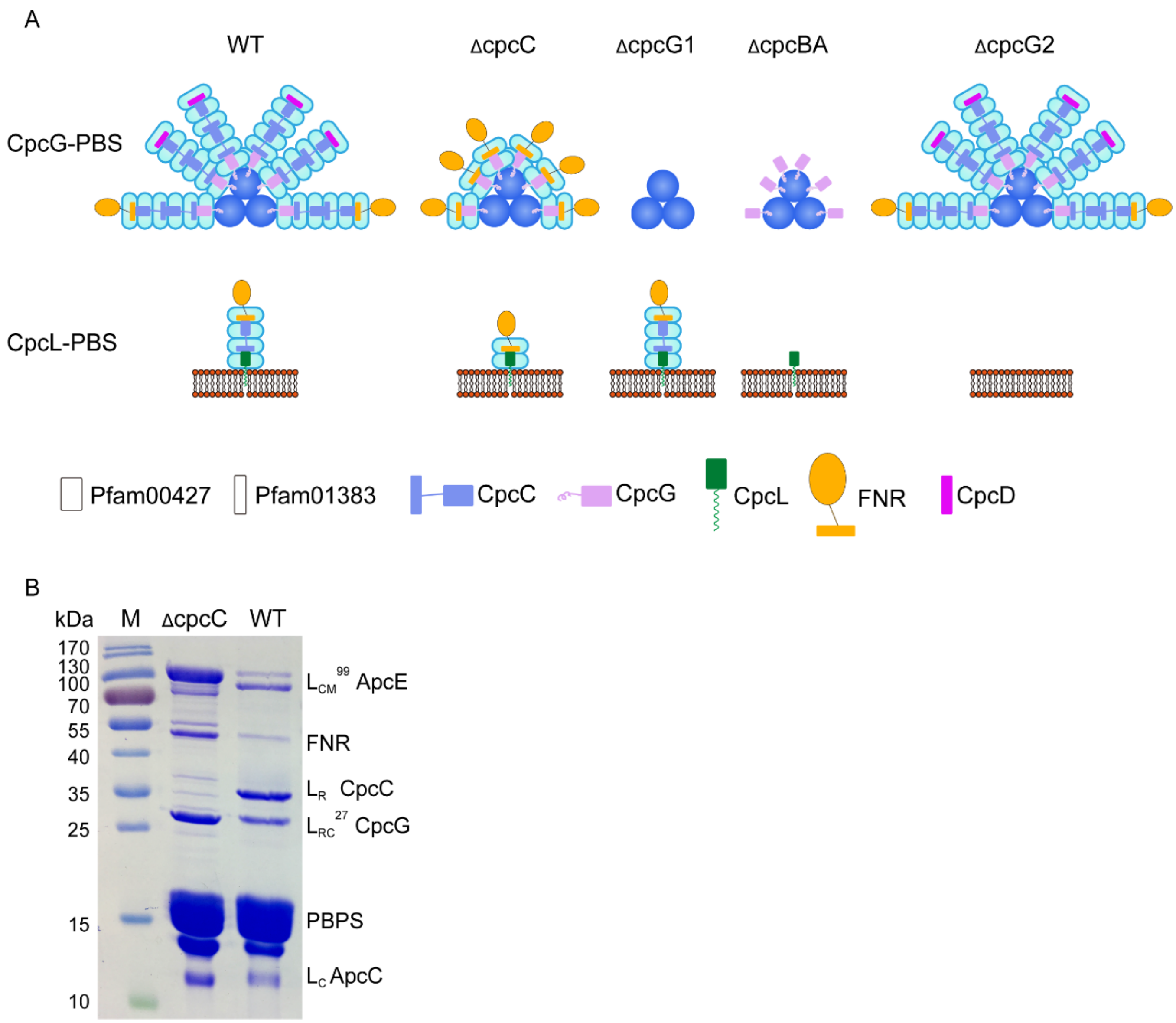

Supplementary Figure S4. Schematic representations of CpcG-PBS and CpcL-PBS in different strains. (A) Schematic drawing of PBS. The CpcG-PBS are shown in upper row and the CpcL-PBS are shown in lower row. Strain names are on the tops of each column. The threes dark blue circles represent the PBS core. Peripheral rods are shown in light blue. The single disc in the rods represents trimers of phycobiliproteins, and the attached FNR are shown in yellow. Only 2 peripheral rods of the CpcG-PBS contained FNR for wild-type based on the estimation by Gomez-Lojero et al. [28]. More FNR are attached to the rods of the *cpcC* mutant. Linker proteins CpcC, CpcG, CpcL and CpcD are shown in indigo, light pink, green and violet colors. Two linker domains Pfam00427 and Pfam01383 are represented by broad rectangle and narrow rectangle, respectively. CpcL-PBS attached to thylakoid membranes by CpcL in WT, ΔcpcC and ΔcpcG1. (B) SDS-PAGE analysis of the CpcG-PBS from WT and ΔcpcC. The FNR bands at 45 kDa position in both WT and ΔcpcC lanes can be observed with more FNR present in CpcG-PBS from ΔcpcC, agreeing with Gomez-Lojero et al. [28].

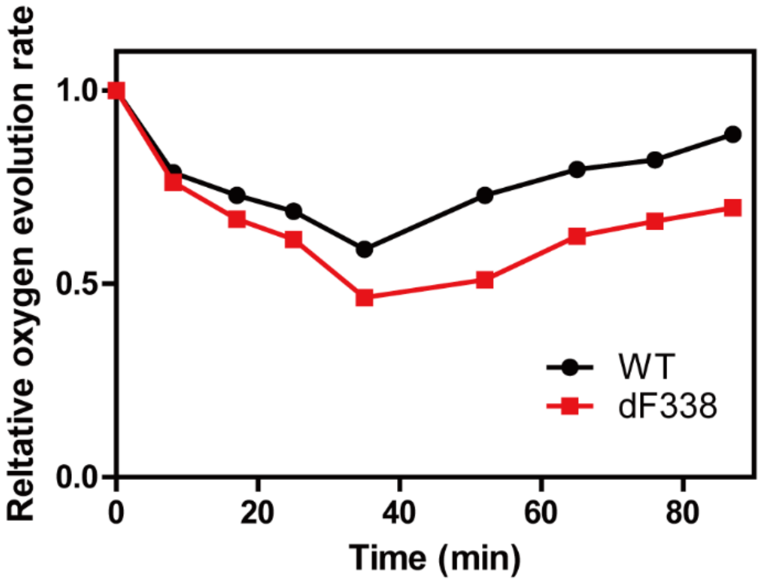

Supplementary Figure S5. Photoinhibition and recovery of wild-type (WT) and dF338. Cultures (Chl concentration at 5 μg ml^{-1}) of WT (black line) and dF338 (red line) were exposed to a strong light at intensity of 1300 μmol photons m^{-2} s^{-2} for 35 min at 35°C before they were exposed to a low light at intensity of 200 μmol photons m^{-2} s^{-2}. Oxygen evolution rates were measured as indicated. The data are presented after normalization of the initial rates before photoinhibitory treatment and they were the averages of three measurements.

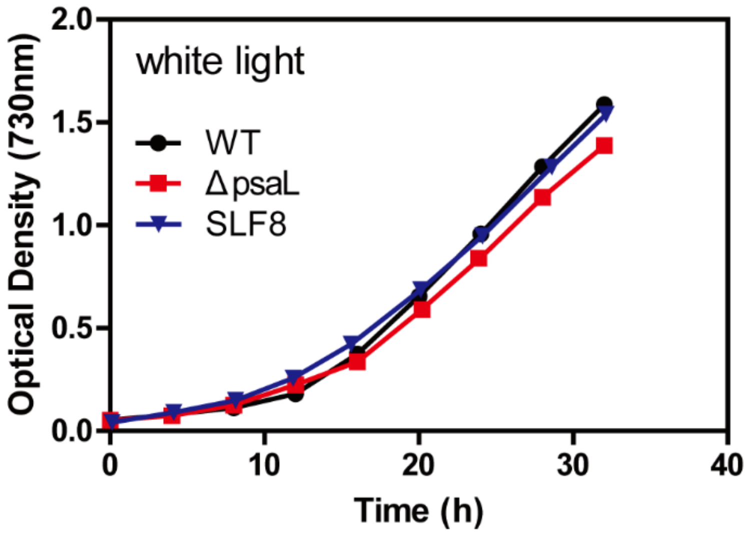

Supplementary Figure S6. Growth curves of strains under photoautotrophic condition. Wild-type (WT, black) and ΔpsaL (red)and SLF8 (blue) were grown at 35℃ under white light condition, which was provided with cool white fluorescent light at 200 µmol photons m^{-2} s^{-2}.

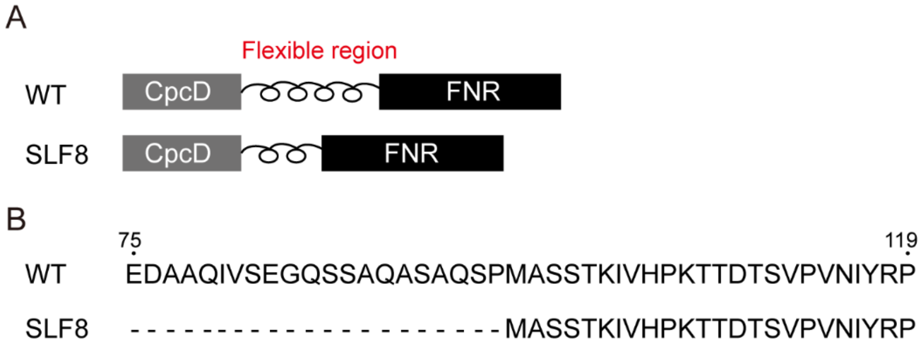

Supplementary Figure S7. Molecular characterization of wild-type (WT) and SLF8. (A) Schematic drawing of FNR are shown for WT and SLF8. The flexible regions of FNR are shown as spiral curves and this region was shortened in SLF8. (B) Sequence alignment for the flexible regions of FNR (residues 75 to 119) between WT and SLF8. Deleted residues are shown with dash lines.

Supplementary Table S1. Sequences of the oligonucleotides primers.

Primer Name	Nucleotide Sequence	Primer Name	Nucleotide Sequence
P1	tccattgatgtcaaatttcc	P2	gcgtgaagcttatcgatacccgggcgataaatattaactg
P3	cagttaatatttatcgcccgggtatcgataagcttcacgc	P4	ccggcatttaaacattacatcttggtcggtcatttcgaac
P5	gttcgaaatgaccgaccaagatgtaatgtttaaatgccgg	P6	gatttttgtcgaagaagccattttatctcctacttatattttg
P7	caaaatataagtaggagataaa atggcttcttcgacaaaaatc	P8	ttagtaaacttccacatgcc
P9	agtgccgatgagcgacagaa	P10	agcaattgattacgagtcag
P11	caaaatataagtaggagataaaatgtacggtatcactagcac	P12	gatttttgtcgaagaagccattgcgggacggatactaacg
P13	cgttagtatccgtcccgcaatggcttcttcgacaaaaatc	P14	atgtaatgtttaaatgccggcagacg
P15	tgagtgcttgcggcagcgtgttagtaaacttccacatgcc	P16	ggcatgtggaagtttactaacacgctgccgcaagcactca
P17	cggggtgggcgaagaactccagcat	P18	atacgaccgctgttcaagta
P19	cgtctgccggcatttaaacattacattcctagctcacccgatgcga	P20	atgctggagttcttcgcccaccccgcgagaagcattagtagaatg
P21	tggcctatttccagatggcc		
